# An Atypical Presentation of Sporadic Creutzfeldt–Jakob Disease in the Setting of Chronic Alcohol Use Disorder

**DOI:** 10.1155/crnm/1332654

**Published:** 2025-10-29

**Authors:** Coral Holt, Alia T. Sadek, Jordan Woodard, Rhett Grinstead

**Affiliations:** ^1^Department of Medical Education, University of South Carolina School of Medicine Greenville, Greenville, South Carolina, USA; ^2^Department of Internal Medicine, Prisma Health Upstate, Greenville, South Carolina, USA

**Keywords:** alcohol use disorder, Creutzfeldt–Jakob disease, Wernicke encephalopathy

## Abstract

Sporadic Creutzfeldt–Jakob disease (CJD) is a rare but universally fatal condition with cardinal symptoms of rapidly progressive dementia and myoclonus. Wernicke encephalopathy (WE) is a reversible condition often presenting with the triad of altered mental status, ophthalmoplegia, and ataxia. Previous case reports have demonstrated overlap in the clinical features, imaging, and laboratory testing of CJD and WE. Here, we present the case of a 60-year-old female who presented with prominent aphasia and ataxia, lacking myoclonus and specific electroencephalogram (EEG) findings of CJD. Our patient's presentation was initially most suspicious for WE in the setting of alcohol use disorder, though maintaining a broad differential prompted extensive workup. Brain magnetic resonance imaging (MRI) was a key factor in distinguishing this case, as there were no lesions in the thalami, mammillary bodies, or periaqueductal gray matter, areas strongly associated with WE. Cerebrospinal fluid (CSF) testing for RT-QuIC, T-Tau protein and 14-3-3 GAMMA, and ultimately autopsy confirmed the diagnosis of sporadic CJD. We compare the clinical features, MRI, and EEG findings of our patient to those of similar cases, recognizing common areas of involvement that are also affected in WE. This case brings further attention to the variable presentation and clinical overlap of CJD with other neuropsychiatric diseases. We therefore endorse strong recommendations for maintaining a broad differential in patients presenting with nonspecific neurological complaints and promptly evaluating with MRI to better localize the affected areas.

## 1. Introduction

Sporadic Creutzfeldt–Jakob disease (CJD) is a rare but universally fatal condition affecting one person per million in the world and 350 cases in the United States annually [1]. Cardinal symptoms of CJD include rapidly progressive dementia and myoclonus, although ataxia, seizures, and personality changes can also occur [1]. CJD is definitively diagnosed via brain autopsy, though cortical ribboning on magnetic resonance imaging (MRI) and cerebrospinal fluid (CSF) analysis for real-time quaking-induced conversion (RT-QuIC), t-tau protein, and 14-three to three GAMMA support a premortem diagnosis of CJD in suspected cases [1]. Wernicke encephalopathy (WE), a reversible neurologic disorder resulting from thiamine deficiency most commonly in the setting of chronic alcohol use or malnutrition, presents similarly to CJD with the classic triad of altered mental status, ophthalmoplegia, and ataxia [2]. Here, we report a case of sporadic CJD with an atypical presentation further obscured by a history of chronic alcohol use disorder (AUD).

## 2. Case Report

**Figure 1:**
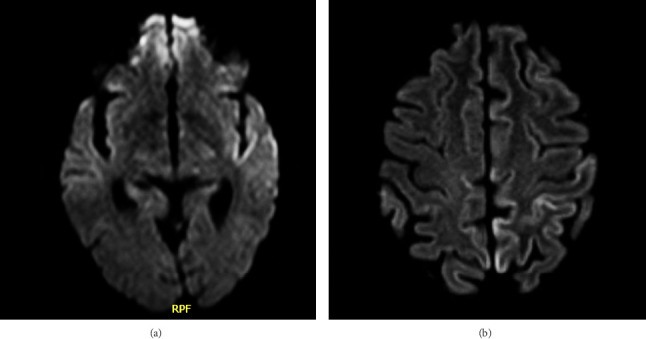
(a) MRI from initial presentation showing mild restricted diffusion and T2/FLAIR hyperintense signal involving the left insula and anterior temporal lobe cortex. (b) Repeat MRI 4 weeks later showing abnormal diffusion and T2 FLAIR signal in the cerebral cortex bilaterally compatible with cortical ribboning.

A 60-year-old female with a past medical history significant for hypertension, hyperlipidemia, insomnia, major depressive disorder, and AUD presented to the emergency department with her husband for a 5-day history of expressive aphasia and a subacute history of confusion. They described her symptoms as worsening slurred speech, confusion, and memory loss. She denied prior similar symptoms, history of neurologic disorders, recent substance use, or trauma. On physical exam, her vital signs were stable, and she was alert and oriented. She had an National Institute of Health Stroke Scale (NIHSS) of 3 due to significant dysarthria and aphasia; otherwise, there were no focal neurologic findings. Basic laboratory work-up was negative including complete blood count (CBC), comprehensive metabolic panel (CMP), urinalysis, urine drug screen, and electrocardiogram (ECG). Computed tomography (CT) of the head also showed no acute findings. The patient was admitted to rule out cerebrovascular accident, and antiplatelet and statin therapy were initiated. Repeat NIHSS on admission was 0 as the patient's symptoms quickly resolved. Echocardiogram and CT angiography of the head showed no abnormalities. MRI of the brain showed mild restricted diffusion and T2/FLAIR hyperintense signal involving the left insula and anterior temporal lobe cortex (Figure 1(a)) which could indicate ischemia, encephalitis, or seizure. Following neurology consultation, it was determined that the patient's aphasia was most likely due to a cerebrovascular incident and that her subacute onset of confusion was an early manifestation of a neurocognitive disorder. The patient was discharged the following day on antiplatelet and statin therapy with expedited outpatient neurology follow-up.

When the patient was seen by outpatient neurology the day after discharge, she reported intermittent episodes of confusion, difficulty hearing, and hesitancy in speech but no deficits in fund of knowledge, written communication, or activities of daily living. Outpatient MRI and electroencephalogram (EEG) were ordered; however, the patient was not able to undergo these tests before she returned to the emergency department 4 weeks after her first admission for a 6-day history of ataxia. History was obtained solely from the patient's husband as she was now unable to participate due to severe aphasia and confusion. Reportedly, her ataxia started when she used the restroom and came back acutely altered. The patient's aphasia and confusion from the previous admission had also worsened, now unable to communicate even with writing, agitated, and lethargic. Her husband reported that she had been drinking much more alcohol than usual over the last month, but she had not drunk any alcohol in the last week. On physical exam, her vital signs were stable, but she was unable to follow commands for complete neurologic assessment. Repeat CT head showed no acute abnormalities, and initial work-up revealed a normal CBC, CMP, urine drug screen, urinalysis, ethyl alcohol and salicylate levels, ammonia, thiamine, and ECG. Given high suspicion for WE, empiric IV thiamine treatment was initiated at 500 mg three times per day, and she was given valproate for empiric seizure prophylaxis. She was then admitted for further work-up and neurology consultation. Subsequent studies for thyroid function, antinuclear antibodies, human immunodeficiency virus, paraneoplastic antibodies, B12, C-reactive protein, erythrocyte sedimentation rate, and bacteremia were all unrevealing. However, repeat brain MRI showed abnormal diffusion and T2 FLAIR signal in the cerebral cortex bilaterally, demonstrating progressive cortical ribboning when compared to her prior MRI (Figures 1(a) and 1(b)). There was symmetrical involvement of the temporal, parietal, and insular regions and asymmetric involvement of the left occipital and posterior frontal lobes. Long-term EEG showed generalized slowing consistent with severe encephalopathy. It also revealed frontal intermittent rhythmic delta activity (FIRDA), indicating nonspecific cerebral dysfunction. Considering these findings, it was thought that the most likely cause of her acute encephalopathy was either prion disease or autoimmunity. Lumbar puncture was performed the day after admission, and CSF studies yielded normal glucose, no organisms, no oligoclonal bands, and negative autoimmune and infectious encephalitis panels. However, her CSF was remarkable for mildly elevated protein at 61.1 mg/dL (normal: 15.0–45.0 mg/dL). Her CSF samples were then sent to the National Prion Disease Pathology Surveillance Center, and 10 days later, it resulted in a positive RT-QuIC, t-tau protein of 3460 pg/mL (normal: 0–1149 pg/mL), and 14-3-3 GAMMA level of 19,838 AU/mL (normal: < 30–1999 AU/mL) (Table 1), indicating a 98% likelihood of prion disease. The patient's symptoms continued to progress requiring urgent intubation and transfer to the intensive care unit where she remained for 7 days. Long-term EEG began to show triphasic waves in addition to generalized slowing, indicative of nonspecific encephalopathy, and her family ultimately elected to transition to hospice care. Unfortunately, her death came only 3 days after the probable diagnosis of prion disease. Autopsy showed positive Western blot analysis and positive hematoxylin and eosin and immunohistochemical staining, confirming the diagnosis of sporadic CJD. No pathogenic variant was detected on PRNP gene sequencing analysis.

## 3. Discussion

Here, we describe a case of sporadic CJD that presented with ataxia, confusion, and aphasia over the course of 1 month. Typically, CJD presents with rapidly progressive dementia and myoclonus; gait instability and ataxia are less likely seen but have been reported [3, 4]. As our patient lacked the classic signs of CJD, she was initially suspected to have WE in the setting of AUD and was treated empirically with thiamine. However, the differential was kept broad as she lacked the ocular abnormalities usually seen in WE, and the early presence of aphasia would be atypical. In addition, MRI lesions did not involve areas typically associated with WE, including the thalami, mammillary bodies, or periaqueductal gray matter, providing reasonable suspicion for an alternative diagnosis. These clinical abnormalities prompted the large-scale work-up which ultimately led to the diagnosis of CJD.

In addition to an atypical clinical presentation, our patient also lacked classic findings of CJD on EEG. Periodic sharp wave complexes (PSWCs) are seen on EEG in two-thirds of CJD cases and typically appear in the middle to late stages of the disease [5]. Our patient's initial EEG findings were only remarkable for diffuse slowing, which can be seen in both WE and CJD, and FIRDA, which is similarly nonspecific though it can be seen in early CJD [6]. Her later EEG also showed triphasic waves which are typically seen in toxic-metabolic encephalopathies though they can be seen in severe cases of WE [7].

On MRI, our patient did display characteristic findings consistent with CJD. Cortical ribboning seen on diffusion weighted, or FLAIR imaging has a 98% sensitivity and 93% specificity for CJD [1]. Sporadic CJD typically shows hyperintensities in the cortical gray matter, basal ganglia, and thalamus [1]. Our patient's last MRI demonstrated symmetric cortical involvement in the temporal, parietal, and insular regions and asymmetric cortical involvement in the left occipital and posterior frontal lobes. The asymmetric involvement of the left posterior frontal lobe consistent with Broca's area could explain our patient's prominent expressive aphasia. Similarly, one previous case reported CJD presenting as primary progressive aphasia with MRI showing similar left-sided hemispheric involvement [8]. It is important to note that although our patient's MRI findings pointed strongly toward CJD, cortical ribboning has been reported in cases of WE initially mistaken for CJD due to diffusion restriction and FLAIR hyperintensity in the bilateral thalami on MRI [9]. Nevertheless, our patient's MRI did not show any lesions in the thalami, mammillary bodies, or periaqueductal gray matter which was a key factor that ultimately led the team away from a diagnosis of WE.

The challenge of diagnosing CJD persists due to the inability to confirm the diagnosis premortem. Definitive diagnosis relies on postmortem brain autopsy to identify classic spongiform degeneration of the cortex, caudate nucleus, thalamus, putamen, and cerebellum [1]. Only genetic and molecular testing from our patient's autopsy was available, so gross description consistent with these changes is unknown. However, cases with similar clinical presentations concerning for WE have demonstrated distinguished patterns of brain area involvement on autopsy. One case reported a patient with AUD who presented with tremor, ataxia, memory loss, confabulation, and severe insomnia without myoclonus or PSWC typical of CJD [10]. Autopsy showed significant spongiform changes in the mammillary bodies and thalamus, explaining the patient's clinical presentation consistent with WE [10]. Another case reported a patient with ataxia, confusion, dementia, and late-stage myoclonus with marked involvement of the cerebello-mammillothalamic system on autopsy [11]. Based on these reports, the atypical presentations of CJD initially concerning for WE may be directly associated with spongiform degeneration of areas classically implicated in WE.

This case brings further attention to the variable presentation and clinical overlap of CJD with other neuropsychiatric diseases. Here, we add to the few number of cases demonstrating such variability and highlight the importance of maintaining a broad differential diagnosis in patients who present with a constellation of neurologic symptoms. Specifically, our case highlights that the location of lesions on MRI can be a significant differentiating factor when CJD mimics other neurological syndromes such as WE. This nuance can allow for a more timely diagnosis which is instrumental in ruling out other causes, providing answers to patients and families, and offering appropriate supportive care.

## Figures and Tables

**Table 1:** CSF analysis from the national prion disease pathology surveillance center for RT-QuIC, t-tau protein, and 14-3-3 GAMMA.

Value	Result	Reference range
RT-QuIC	Positive	Negative
T-tau protein	3460 pg/mL	0–1149 pg/mL
14-3-3 GAMMA	19,838 AU/mL	< 30–1999 AU/mL
